# Non-Communicable Diseases, Longevity, and Health Span: A Hong Kong Perspective

**DOI:** 10.3390/ijerph22030359

**Published:** 2025-02-28

**Authors:** Jean Woo, Michael Marmot

**Affiliations:** 1 CUHK Institute of Health Equity, The Chinese University of Hong Kong, Hong Kong SAR, China; m.marmot@ucl.ac.uk; 2 Department of Medicine & Therapeutics, The Chinese University of Hong Kong, Hong Kong SAR, China; 3 Institute of Health Equity, University College London, London WC1E 7HB, UK

**Keywords:** total life expectancy at birth, frailty, intrinsic capacity, health span, dependency, health inequality, social determinants of health

## Abstract

The health of different countries evolves in parallel with their economic development. Communicable diseases play a more prominent role in low-income countries compared with high-income countries, while non-communicable diseases (NCDs) have become dominant in developing and developed economies. This transition has been accompanied by public health efforts to prevent NCDs, resulting in improvements in total life expectancy at birth (TLE). It is recognized that health determinants are not confined to healthcare systems, but that social determinants play a key role in health inequalities. The rapid increase in life expectancy at birth has led to increasing numbers of older adults, where health inequalities are accentuated. The current UN Decade of Healthy Aging calls for a life-course approach to building intrinsic capacity, maintaining function, and avoiding dependency with age instead of avoiding mortality and morbidity. TLE in Hong Kong is one of the highest in the world as a result of public health preventive efforts and an essentially free hospital system. However, the increase in TLE has not been accompanied by the same magnitude of increase in health span, resulting in an increasing dependency burden. Geography, culture, dietary and smoking habits, physical activity, urban planning, and neighbourhood cohesion are some of the social determinants affecting TLE and also health inequalities. With population ageing, it would be appropriate to focus on the social determinants affecting health span to maintain functional independence rather than prolonging life. A whole-of-society response instead of relying solely on the adaptation of health and social care systems would be needed, together with more nuanced metrics to measure health span.

## 1. Introduction

We are at the start of a third revolution in global health. The first, which is still active, is disease control through specific disease programs, such as malaria, tuberculosis (TB), human immunodeficiency virus (HIV) and now non-communicable diseases. The second revolution, which is also still active, aims to develop effective health systems and reduce risky health behaviours. The commendable goal of Universal Health Coverage [1] stems from the goal of building effective health systems and still has a great deal further to go, including incorporating technological advances.

The third revolution aims to improve health and health equity through action on the social determinants of health. This comes from the recognition that the key drivers of health, and health inequalities, lie outside the health care system. Inequalities in health—such as life expectancy gaps of 40 years between countries [2] and 20 years or more within countries such as the United Kingdom [3]—will not be prevented solely by specific disease control measures, behaviour change programmes or further development of health care systems.

## 2. Discussion

Health inequalities arise because of inequalities in the social, environmental, political and economic conditions in which people are born, grow, live, work and age. Over the last twenty years, evidence about the effectiveness of the social determinants approach has grown significantly. This has led to increasing prioritization and understanding of the drivers of health inequalities and the implementation of approaches to tackle them through action on social determinants.

The World Health Organization (WHO) Commission on Social Determinants of Health (chaired by Prof. Sir Michael Marmot) has brought together and analysed considerable evidence on the social determinants of health. Its 2008 report, *Closing the Gap in a Generation* [4], stated that “social injustice is killing on a grand scale”. The report presented the moral case for action on health equity and set out evidence-based recommendations for action to close the health gap within a generation. This work is built on research in social epidemiology, child development, education, sociology, biology, and economics, based largely in North America and Western Europe, and identifies the ways in which social, environmental, political and economic conditions cause disease, disability and increased mortality risk worldwide.

Health and social inequities are accentuated among older people, as they face both inevitable physical and cognitive declines in a setting of health and social care systems that are not designed to meet their complex needs. At the same time, exiting the workforce causes problems with income security, social protection systems are not always adequate, housing conditions may harm physical and mental health and environmental factors in more deprived areas also pose risks to health. Many older people are physically and socially isolated and face disconnection from society, the consequences of which have been well documented to contribute to loneliness. The latter has both physical and psychological health consequences, which have been compared with the risk to health of smoking 15 cigarettes a day [5].

But inequalities start early in life; evidence shows that the conditions in which babies are born and grow and experiences during childhood and adolescence have profound impacts on health at older ages. Thus, inequalities in early life relate directly to inequalities in health and a range of other social, economic and political inequalities later in life. They also have an impact on health and other life opportunities during the early years and adolescence. There is now a substantial evidence base establishing the importance of early years and education for health and there is a good level of knowledge about which programmes and policies work best to reduce inequalities in early years, which can reduce inequalities throughout life [6].

Japan has blazed a trail in Asia. It went from a relatively poor, unhealthy post-war country through rapid social and economic development to having the world’s longest life expectancy. By 2024, post-COVID, Hong Kong had overtaken Japan—with an average life expectancy of 88.3 years for women, and 83.0 years for men. Singapore and the Republic of Korea are not far behind. To put this success in context, life expectancy for women is 83.0 years in the UK, and 82.0 years in the USA; for men, the figures are 79.5 years in the UK and 77.0 years in the USA. The US figures are, on average, 5 years behind Hong Kong. Elsewhere, it has been observed that total life expectancy at birth can be used to track health inequalities when disaggregated between and within countries [2]. It is uncertain whether this remarkable increase in TLE is accompanied by diminishing health inequalities. A rapid decline in infant mortality to the lowest in the world (for Hong Kong) as well as decline in mortality from non-communicable diseases (especially cardiovascular diseases) are major contributors to the increase in TLE. Mortality data in Hong Kong were examined from 1950 to 2016 and compared with 18 high-income countries. From 1979 to 2016, Hong Kong was shown to accumulate survival advantage over other countries, attributable to a decline in cardiovascular and cancer mortalities. It was hypothesised that this increase in longevity compared to other high-income countries was due to a combination of economic prosperity as well as effective implementation of government policies against smoking [7], in the context of an essentially free health care system.

Health and social care systems clearly have an important role in the prevention and management of NCDs. There is a social safety net in Hong Kong, which ensures that healthcare in hospitals is essentially free and subsidised by the government. The system is modelled after the UK’s National Health Service and social welfare. The UK also has a free primary care system, which Hong Kong does not have. Yet, life expectancy is lower in the UK. Japan, Korea, and Singapore have an insurance-based healthcare system, unlike the UK; again, the life expectancy is higher in these countries. The US also has an insurance-based healthcare system, but the life expectancy is lower than that of the UK and these Asian countries. Such observations serve to highlight the importance of social determinants of health, outside of health and social care systems, in influencing life span and health span.

There is a paucity of data documenting social determinants of health, such as socioeconomic position, physical and social environment, changing trends in lifestyle behaviours, and cultural and other societal characteristics, in addition to health and social systems and policies. Hong Kong has huge wealth inequalities (Gini Coefficient 0.539) and inequalities in which people live, work and age. These inequalities are expected to drive health inequalities, an association well documented all over the world. However, in Hong Kong and other Asian countries, these socioeconomic inequalities have not resulted in reduced average life expectancy. The question remains whether there are ameliorating factors that are cultural/social/physical environmental in nature, or whether health inequalities are merely undocumented. An increase in TLE may also be occurring at the expense of rising levels of dependency, which may also be subject to inequalities. How far is Hong Kong from a life-course approach to healthy ageing that focuses on creating maximal intrinsic capacity from birth to peak young adulthood and then maintaining and slowing decline in intrinsic capacity thereafter? What are the social determinants of healthy ageing?

In line with the World Health Organization’s (WHO) proposed measurement of health that is not based on mortality rates but on measures of functioning [8], indicators of health inequalities would need to include such measures when addressing the question of whether this remarkable increase in TLE is occurring in the context of diminishing health inequalities. A recent survey of 183 WHO member states highlighted the widening gap between life span and health span [9].

Recent data in Hong Kong suggest that there is a rising trend in daily activity deficits as well as frailty among recent cohorts of older people, with increasing life expectancy projected to increase the absolute number of dependent older people [10,11,12]. Using the Global AgeWatch Index [13] approach comparing 97 countries and territories, Hong Kong ranks as number one in health based on life expectancy at birth, yet it is ranked at seventy-nine for psychological health, and sixty-one for income security. Social determinants have received little attention because health and social systems are still based on a disease-based approach, which pays little attention to the social determinants of health. Hong Kong is divided into 18 districts for government administrative purposes, and these differ in terms of various socioeconomic and social characteristics such as median income, educational level, and indices of deprivation [14]. A current survey among community-dwelling older people in Hong Kong showed extensive inequalities in levels of prefrailty and frailty that vary by district and education level [15]. For example, the Southern district had the highest proportion of people with pre-frailty/frailty (77.7%) and the Central/Western district had the lowest (45.8%), while those of other districts ranged between approximately 58.7% (Kwai Tsing) and 70.6% (Tsuen Wan). Likewise, the rates of pre-frailty/frailty varied by education level: those with less education (62.8–71.9%) were more likely to be pre-frail/frail compared to those with higher education (54.4–56.2%). An ongoing territory-wide initiative to promote the WHO’s Age Friendly City principles showed that the rating of ‘age-friendliness’ by district follows a social gradient according to socioeconomic level and that ‘age-friendliness’ characteristics correlate strongly with self-rated health, as well as a sense of community [16].

Hong Kong is an ageing society, where the health and social care systems are concerned with coping with the increasing prevalence of chronic diseases and their associated dependencies, with the accompanying cost implications and need for care staff. A survey was carried out using the database of Elderly Health Centres of the Department of Health from 2001–2012, with over 90,000 individuals from the community-dwelling population older than 65 years. A worrying trend shows that today’s older people have higher levels of frailty than previous generations [11]. During this period, the number of years lived with physical impairment at 65 years increased, more so in women; however, the increase in life-years with cognitive impairment surpassed that of physical impairments. The accompanying increase in the number of care workers needed and costs may be estimated and projections made if the trend continues. Unfortunately, there is no regularly collected data by the government to inform policies [12].

A recent series of reports compiled by the Institute of Health Equity at University College London and the Institute of Health Equity in the Chinese University of Hong Kong documented clear social gradients in various health outcomes. Low household monthly income is associated with poor self-rated health and prevalence of chronic disease as well as multimorbidity [17]. It is clear that the increasing TLE in Hong Kong does not represent diminishing health inequalities. The high TLE is likely a result of an essentially free health care system with an emphasis on promoting maternal and child health, a hospital-based secondary and tertiary care that is able to prolong life, and a pro-poor social welfare system.

There are many other factors that may contribute to the high TLE, despite the existence of health inequalities measured using other outcomes. Hong Kong is an example of a compact urban city, with a high population density. It occupies 1104 sq km, but half of this land consists of country parks that are not zoned for building development. Essentially, it is a vertical city where residents live in high-rise flats in close proximity to each other, with easy access to transport hubs, wet markets selling fresh fruits, vegetables, seafood and meat at a cost lower than most supermarkets. Hence, a ‘healthy diet’ is affordable to all. There is a pervasive culture of traditional Chinese medicine that emphasises certain dietary and physical activities incorporating mind–body exercises in maintaining health over treatment of diseases. At the same time, parks and beaches are short distances away from the built environment and easily accessible by public transport, which is subsidised for those aged 60 years and over. There is easy and convenient access to all services such as banks, clinics, and various social gathering venues. For sudden illnesses, ambulance transfers to hospitals are efficient. There is also a low crime rate. Due to its small size, the frequency of natural disasters is low. However, on the negative side, there are issues with workplace stress, the high cost of home ownership and very small living spaces, which have been shown to have adverse health outcomes.

Social isolation and loneliness are medical issues [5]. It might be expected that in such a dense living environment, the prevalence may not be high. Among older adults, insufficient care, a growing distance between them and society and their disintegrating identity in society are primary sources of their loneliness. Loneliness may be mitigated by higher levels of neighbourhood social cohesion. Older people with a lower socioeconomic position are at greater risk of social isolation. There is a lack of data regarding the overall impact of social isolation and loneliness in such a compact city on health inequalities.

Great wealth inequality in a collectivistic society can result in a strong response from the wealthy in supporting those who are disadvantaged, in the form of charitable foundations. The city has a very strong philanthropic culture that dates back to various missionary and Buddhist groups over one hundred years ago. Currently, there are over 10,000 registered charities in Hong Kong [18].

Using the World Health Organization’s metric of healthy ageing throughout the course of life that emphasises function over mortality [19], Hong Kong does not perform so well, as indicated by the rising prevalences of frailty and dependency [10,11]. There may be a growing number of centenarians, but many reside in residential care homes and have various levels of dependency. A large Chinese study used five criteria of successful ageing to study centenarians in the Chinese Longitudinal Healthy Longevity survey: absence of disability, lack of cognitive impairments, presence of life satisfaction, social engagement, and few medical conditions. Only 9% of men and 5% of women among the centenarians met all five criteria, while 1% did not meet any of the criteria [20]. There has been no similar study among the Chinese population in Hong Kong, but this finding illustrates how TLE may not be a good measure of health outcome when TLE has increased to a certain level in monitoring health inequalities. Some multi-domain indicators such as intrinsic capacity, as proposed by the WHO in the current UN Decade of Healthy Aging, would be more appropriate. A recent review examined the social determinants of healthy ageing using intrinsic capacity as a measure [21].

## 3. Conclusions and Future Directions

As a result of the ‘second revolution’ in global health that focuses on health systems and the prevention of risky behaviours, an increasing TLE has been and is still being achieved. Improvements in the social determinants of health: education, income and wealth, and better social services have also contributed. For the ‘third revolution’, which takes into account a host of social determinants, a more nuanced metric would be desirable that places importance on functioning, rather than mortality, or health span. In this regard, it is doubtful whether Hong Kong can claim to be one of the world leaders, although the whole of society would need to strive towards achieving this goal. Therefore, we must focus on developing strategies that adopt a life-course approach towards improving health span in addition to TLE. The adaptation of health and social care systems may yield results in the short term, but mainly in extending life span by reducing mortality from NCDs, but not necessarily in increasing health span. A life-course approach involving the whole of society dealing with social determinants of health outside of health care systems would be needed.

To accompany this goal, it is necessary for governments to collect regular age and sex disaggregated data, not only on chronic diseases and mortality, but also on measures of physical and cognitive functioning or dependency as well as quality of life, stratified by some deprivation measures. While Hong Kong has many geographic and wealth advantages, there are still extensive unmet needs among older adults, particularly regarding timely low-cost primary care, increasing dependency among those living alone or only with a spouse who requires health and social care, as well as housing affordability. Promoting awareness of such needs by first documenting them and then proposing new models for integrated community care such as the model proposed by the WHO Integrated Care for Older People (ICOPE) that uses a step care model where district-based volunteers may play a role in the first step of identifying unmet needs followed by individualised action plans. It would also be important to carry out prospective studies linking social determinants in earlier stages of the life-course trajectory such as early life, adolescent, and working environment, as well as later life, with health span and healthy life expectancy, along with interventions that mitigate social gradients in these indicators. Government policymakers, community service providers, employers, educators, and healthcare workers in institutions must be aware of the importance of these findings and work together to reduce inequalities in healthy ageing.

## Data Availability

No new data were created or analyzed in this study. Data sharing is not applicable to this article.

## Glossary

*Total Life expectancy:* duration of life from birth to death

*Health span:* health-adjusted life expectancy whereby years of life are weighted by health status

*Healthy life expectancy:* estimates of healthy longevity consisting of a summary indicator of health and quality of life
